# Genome Sequence Resource of *Fusarium graminearum* TaB10 and *Fusarium avenaceum* KA13, Causal Agents of Stored Apple Rot

**DOI:** 10.1094/MPMI-03-22-0069-A

**Published:** 2022-12-13

**Authors:** Mladen Petreš, Jovana Hrustić, Nataša Vučinić, Li-Jun Ma, Dilay Hazal Ayhan, Mila Grahovac

**Affiliations:** 1 University of Novi Sad, Faculty of Agriculture, Trg Dositeja Obradovića 8, Novi Sad, Serbia; 2 Institute of Pesticides and Environmental Protection, Banatska 31B, Belgrade, Serbia; 3 University of Novi Sad, Faculty of Medicine, Department of Pharmacy, Hajduk Veljkova 3, Novi Sad, Serbia; 4 University of Massachusetts Amherst, Department of Biochemistry and Molecular Biology, Amherst, MA 01003, U.S.A.

**Keywords:** apple, *Fusarium avenaceum*, *Fusarium graminearum*, genome, genome assembly, plant-microbe interaction, postharvest pathogen, secondary metabolites

## Abstract

The filamentous fungus *Fusarium graminearum* is a well-known cereal pathogen and *F. avenaceum* is a pathogen with a wide host range. Recently, both species were reported as causal agents of apple rot, raising concerns about postharvest yield losses and mycotoxin contamination. Here, we report genome assemblies of *F. avenaceum* KA13 and *F. graminearum* TaB10, both isolated from fruits with symptoms of apple rot. The final *F. avenaceum* KA13 genome sequence assembly of 41.7 Mb consists of 34 scaffolds, with an N_50_ value of 2.2 Mb and 15,886 predicted genes. The total size of the final *F. graminearum* TaB10 assembly is 36.76 Mb, consisting of 54 scaffolds with an N_50_ value of 1.7 Mb, and it consists of 14,132 predicted genes. These new genomes provide valuable resources to better understand plant-microbe interaction in stored apple rot disease.

## Genome Announcement

The genus *Fusarium* consists of ubiquitous filamentous fungi and includes many plant-pathogenic species. *Fusarium avenaceum* is a pathogen with a wide host range, mostly reported as a causal agent of cereal (Lysøe et al. 2014), potato (Peters et al. 2008), and even crayfish diseases (Makkonen et al. 2013). *Fusarium graminearum*, on the other hand, is a well-known causal agent of one of the most devastating diseases of wheat (Fusarium head blight) and maize (ear rot and stalk rot) (Guo and Ma 2014; Guo et al. 2016). Both species are capable of producing a diverse spectrum of secondary metabolites (Brown and Proctor 2016; Sørensen et al. 2009).

*F. avenaceum* is regularly detected in storages in Europe and the United States (Kou et al. 2014; Sever et al. 2012; Tarlanović et al. 2017; Wenneker et al. 2016) as a causal agent of apple rot. Recently *F. graminearum* has also been detected on diseased apples in Serbia, together with *F. avenaceum* (Petreš et al. 2020). Due to their capability to produce a diverse spectrum of secondary metabolites, including mycotoxins, the occurrence of these pathogens on stored apple could cause not only yield losses but also mycotoxin contamination (Petreš et al. 2018; Sørensen et al. 2009). The availability of whole-genome sequences of *F. graminearum* and *F. avenaceum* isolated from apple can be helpful to understand mechanisms of adaptation to different hosts.

**Fig. 1.**
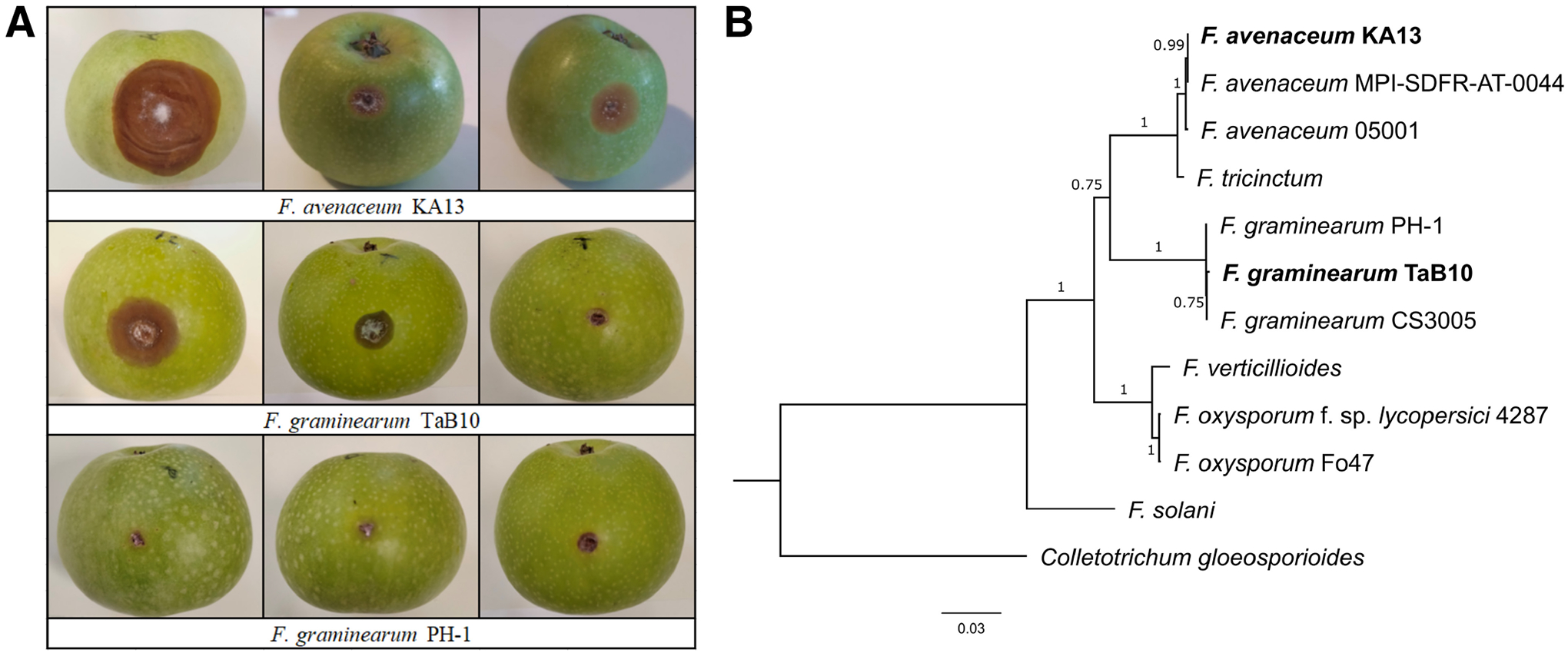
**A**, Disease symptoms. Apples (cultivar Granny Smith) were washed with tap water, were surface-sterilized with 96% ethanol, were injured with a sterile cork borer, and were artificially inoculated with 7-day-old 3-mm mycelium plugs of *Fusarium avenaceum* KA13, *F. graminearum* TaB10, and *F. graminearum* PH-1 cultured on potato dextrose agar. The fruits were incubated at room temperature (21 ± 1°C) for 10 days. Three fruits were prepared for each isolate. **B**, Phylogenetic tree. Protein sequences of the orthologs of 10 conserved and single-copy genes of *F. oxysporum* f. sp. *lycopersici* 4287 (FOXG_00800, FOXG_00887, FOXG_01751, FOXG_07784, FOXG_09267, FOXG_09377, FOXG_02073, FOXG_04212, FOXG_10639, and FOXG_03560) were aligned, using the ClustalW algorithm in MEGA11 (Tamura et al. 2021), multi-sequence alignments were concatenated into a single multi-sequence alignment and the tree was generated via MEGA11, using the maximum likelihood method and JTT matrix-based model (Jones et al. 1992), with 1,000 bootstraps. The scale represents number of substitutions per site.

Here, we present the genome sequences of isolated casual agents of stored apple rot, *F. avenaceum* KA13 (cultivar Idared, locality Kać, Serbia, year of isolation 2012, called FaKA13 hereafter) and *F. graminearum* TaB10 (cultivar Braeburn, locality Tavankut, Serbia, year of isolation 2016, called FgTaB10 hereafter). After artificial inoculation of apple fruits, both strains caused apple rot, while control strain *F. graminearum* PH-1, a pathogen of cereals, did not show pathogenicity (Fig. 1A).

Genomic DNA was extracted from mycelial cultures of FaKA13 and FgTaB10 grown on potato dextrose agar medium (PDA), using the Qiagen DNeasy plant mini kit. Whole-genome sequencing was performed using the Illumina MiSeq platform, with v3 reagents and 2 × 150 cycles with an average coverage of 113× for the FaKA13 genome and 100× for the FgTaB10 genome (Table 1). The quality of the reads was assessed via FastQC version 0.11.5 (Andrews et al. 2010). Trimmomatic version 0.32 (Bolger et al. 2014) was used for read trimming and adaptor removal, with parameters ‘ILLUMINACLIP: TruSeq3-PE-2.fa:2:30:10 TRAILING:10 MINLEN:36′. The initial assemblies were generated using ABySS version 1.5.2 (Simpson et al. 2009). Assembly mapping, cleaning, and polishing were performed using BWA version 0.7.12 (Li and Durbin 2009), SAMtools version 1.3 (Li et al. 2009), and Picard version 2.0.1. GRIDSS version 1.4.1 (Cameron et al. 2017), a structural variant caller, was used to identify links between scaffolds in the initial assemblies. Scaffolding was performed as described by Ayhan et al. (2018). The mitochondrial chromosome sequences were removed from the assemblies. BUSCO v5.2.1 (Manni et al. 2021) was used to evaluate the completeness of the assemblies, using genome sequences. RepeatScout version 1.0.5 (Price et al. 2005) and RepeatMasker version 4.0.5 (Tarailo-Graovac and Chen 2009) were used to identify and mask repetitive elements and AUGUSTUS version 3.3.2 was used to perform *ab initio* gene annotations (Stanke et al. 2006).

The total size of the *F. avenaceum* KA13 assembly is 41.7 Mb, which is comparable to previously published *F. avenaceum* genomes (Lysøe et al. 2014). The FaKA13 assembly contains 34 scaffolds (N_50_ is 2.23 Mb, the largest scaffold length is 3.71 Mb), with a GC content of 48.07%. The total number of the genes is 15,886, while the repetitive sequences comprise 2.37% of the genome sequence. Benchmarking universal single-copy orthology (BUSCO) analysis shows that 97.8% of BUSCOs were complete (single copy: 97.4%, duplicated: 0.4%) while 0.5% were fragmented (Table 1).

The total length of the final *F. graminearum* TaB10 assembly is 36.76 Mb, and it is slightly larger than the *F. graminearum* PH-1 genome (Cuomo et al. 2007). FgTaB10 assembly is comprises 54 scaffolds, with an N_50_ value of 1.67 Mb, and the largest scaffold was 3.68 Mb. There are 14,132 predicted genes. The GC content is 48.3%, while the repetitive contents are 2.59% of the sequence. Complete BUSCO ratio was 97.7% (single copy: 97.5%, duplicated: 0.2%), while 0.2% was fragmented (Table 1).

To determine the capacity for mycotoxin biosynthesis, the secondary metabolite (SM) gene clusters in FaKA13 and FgTaB10 were predicted using SMURF, which identifies four types of SM clusters (dimethylallyltryptophan synthases, polyketide synthases [PKS], nonribosomal peptide synthases [NRPSs], and hybrid PKS-NPRS) (Khaldi et al. 2010) (Table 1). The total number of gene clusters in FaKA13 is 39, while in FgTaB10, the number of predicted gene clusters is 27.

The protein sequences of 10 conserved and single-copy orthologous genes (Zhang et al. 2020) were selected for phylogenetic analysis from eight species of genus *Fusarium* and were rooted on the sequence of *Colletotrichum gloeosporioides* (Fig. 1B). The analysis showed that FaKA13 is grouped within the *F. avenaceum* clade, while FgTaB10 is grouped within the *F. graminearum* clade.

## Figures and Tables

**Table 1.** Summary of genome assemblies^a^

Statistics	FaKA13	Fa05001	FgTaB10	FgPH-1
Sequencing				
Estimated read coverage	113		100	
Read mapping %	98.77		98.55	
Assembly				
Accession	JABCRA010000000	JPYM01000000	JABCRB010000000	AACM02000000
Assembly size (bp)	41,702,554	41,590,745	36,764,292	36,223,641
Number of scaffolds	34	83	54	433
Size of largest scaffold (bp)	3,718,987	4,337,333	3,680,262	473,223
N50 (bp)	2,236,682	1,436,644	1,672,063	184591
N90 (bp)	703,297	424,894	790,537	52752
GC content (%)	48.07	48.47	48.00	48.33
Interspersed repeat content (%)	2.37		2.59	
BUSCO complete (%)	97.8		97.7	
BUSCO fragmented (%)	0.5		0.2	
BUSCO missing (%)	1.7		2.1	
Annotations				
Predicted gene count	15,886	13,217	14,132	11,640
Total predicted secondary metabolite gene clusters	54		39	
Dimethylallyltryptophan synthases	4	4	0	
Polyketide synthases (PKS)	19	27	15	15
PKS-like clusters	2		1	
Nonribosomal peptide synthases (NRPSs)	16	25	11	20
NRPS-like clusters	11		12	
Hybrid clusters	2		0	

a *Fusarium avenaceum* KA13 and *F. graminearum* TaB10 genome assemblies from this study are provided as well as, for reference, the assemblies of the *F. avenaceum* Fa05001 (Lysøe et al. 2014) and *F. graminearum* PH-1 (Cuomo et al. 2007) genomes. Mitochondrial sequences were excluded.

## Data Availability

The sequences for the FaKA13 whole-genome project are deposited at GenBank under accession number JABCRA000000000. The version JABCRA010000000 was used in this study. The deposited Illumina reads are available in the Short Read Archive (SRA) under accession number SRR11662100. The FgTaB10 whole-genome project is deposited at GenBank under accession number JABCRB000000000. The version used in this study is JABCRB010000000. The Illumina reads are deposited in SRA and are available under accession number SRR11665916. The availability of this resource will provide better insight into the plant-microbe interactions between *Fusarium* species and their hosts.
